# Mitro-aortic infective endocarditis on bicuspid aortic valve multicomplicated: a case report

**DOI:** 10.1097/MS9.0000000000000745

**Published:** 2023-05-03

**Authors:** Mohammed Boutaybi, Othmane Doudouh, Charmake Darar Assoweh, Oussama El Abbassi, Nabila Ismaili, Noha El Ouafi

**Affiliations:** a Department of Cardiology, Mohammed VI University Hospital; b Epidemiological Laboratory of Clinical Research and Public Health, Faculty of Medicine and Pharmacy of Oujda, Mohammed First University, Oujda, Morocco

**Keywords:** enterococci, fever, infective endocarditis, splenic infarction

## Abstract

Infective endocarditis (IE) is a rare but serious disease, and despite improvements in diagnostic and therapeutic tools, it remains associated with high mortality. It can develop in a healthy heart, but most commonly in underlying heart disease. The authors discuss a 34-year-old patient, who has presented for 4 months with generalized asthenia, fever, and chills, without any notion of chest pain, arthralgias. On admission, the patient was conscious, hemodynamically, and respiratory stable, the temperature was 38.5°C. The cardiac exam heart revealed a systolic murmur rated 4/6th at the mitral focus. Transthoracic echocardiography showing vegetation on the mitral and aortic valve. Three blood cultures were taken at 1 h intervals, all positive for enterococci, thoracoabdominal and brain computed tomography scan with contrast injection was performed showing, ischemic stroke, aortic coarctation, splenic and renal infarction, mycotic aneurysms in the descending aort. IE can be the cause of several extracardiac manifestations, through vascular and/or immunological phenomena. Embolic complications are the most frequent extracardiac complications and are secondary to septic emboli from the vegetations. The prognosis of IE is worsened by the addition of cardiac and extracardiac complications such as mycotic aneurysms and septic embolic events.

## Introduction

Highlights

- Infective endocarditis (IE) is a rare but serious infection.
- The bicuspid aortic valve is the most common congenital heart disease.
- Embolic complications are the most frequent extracardiac complications of IE.
- Cardiac surgery is performed in 50% of patients who develop IE.

Infective endocarditis (IE) is a rare but serious disease, and despite improvements in diagnostic and therapeutic tools, it remains associated with high mortality. It can develop in a healthy heart, but most commonly in underlying heart disease. Bicuspid aortic valve (BAV) is the most common congenital heart disease and is associated with a higher risk of cardiovascular complications, including IE.

We report a case of mitro-aortic IE on the BAV, multicomplicated, having progressed well after double mitro-aortic valve replacement.

This case report has been reported in line with the Surgical Case REport (SCARE) 2020 criteria^1^, Supplemental Digital Content 1, http://links.lww.com/MS9/A111.

## Case report

**Figure 1:**
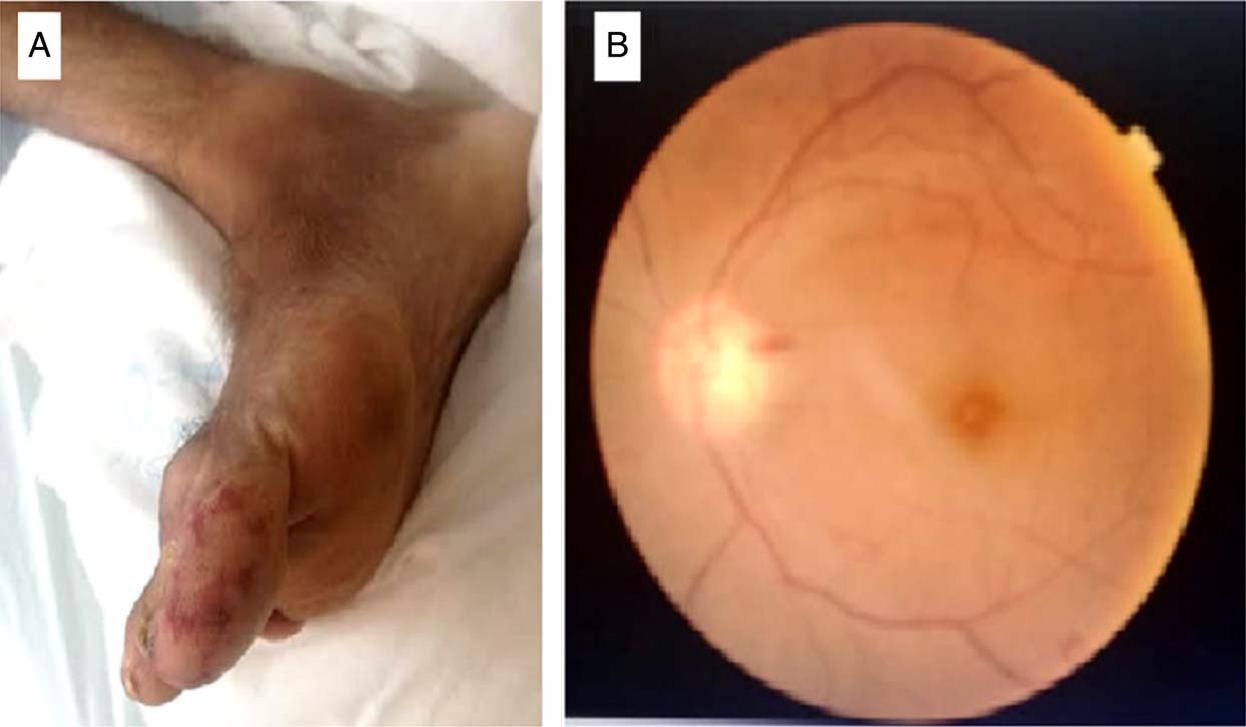
Image that shows purpuric spots on the right dorsal side (A), Fundoscopic Exam showing a roth spot (B).

**Figure 2:**
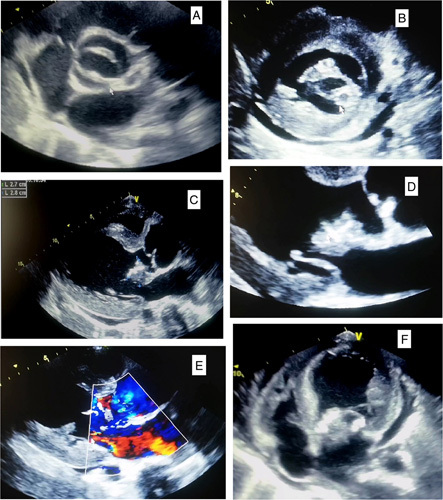
Short axis parasternal section showing bicuspid aortic valve (A),vegetation on the mitral valve (B). Long-axis parasternal section showing vegetation on the mitral and aortic valve (C, and D), mitro-aortic regurgitation (E) four-cavity section showing vegetation on the mitral valve (F).

**Figure 3:**
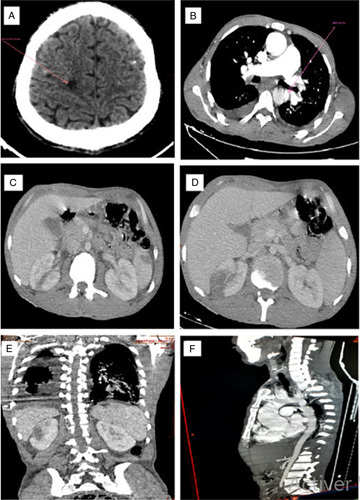
Brain computed tomography (CT) showing poorly limited hypodense right frontal brain spot (A), axial section thoracic CT showing thoracic aortic aneurysm (B), axial section abdominal CT showing splenic infarct (C), renal infarct (D), frontal section abdominal CT showing splenic and renal infarct (E), sagittal section, thoracic CT showing aortic coarctation (F).

This is a 34-year-old patient, who has been presenting for 4 months with generalized asthenia, associated with fever and chills without any notion of chest pain, nor dyspnea or arthralgia. The patient does not report any notion of recent dental care, all evolving in a context of deterioration of the general state with weight loss, which motivated the patient to consult. On admission, the patient was conscious, hemodynamically, and respiratory stable, the temperature was at 38.5°C. The cardiac examination revealed a systolic murmur evaluated at 4/6th at the mitral focus, with purpuric spots on the dorsal face of the right foot (Fig. 1A). The oral cavity examination found a poor oral condition with multiple carries. A 18-leads resting electrocardiogram shows a sinus rhythm with left ventricular (LV) hypertrophy associated with secondary repolarisation disorders. Transthoracic echocardiography (Fig. 2). was performed showing a thickened BAV with filiform vegetation on the ventricular side of the no coronary cusp (video 1, Supplemental Digital Content 2, http://links.lww.com/MS9/A112), dilatation of the ascending aorta (sinus of Valsalva at 49 mm, sinotubular junction at 42 mm), aortic coarctation (Vmax 3. 3 m/s, maximum gradient at 44 mmHg), eccentric aortic regurgitation (regurgitating orifice surface at 1.6 cm² and regurgitation volume at 179 ml). The mitral valve is poorly remodeled with magma of vegetations on the atrial side of the large mitral valve (video 2, Supplemental Digital Content 3, http://links.lww.com/MS9/A113, video 3, Supplemental Digital Content 4, http://links.lww.com/MS9/A114, video 4, Supplemental Digital Content 5, http://links.lww.com/MS9/A115, video 5, Supplemental Digital Content 6, http://links.lww.com/MS9/A116), responsible for a mitral regurgitation (regurgitation volume at 39 ml and regurgitating orifice surface at 0.4 cm²) (video 6, Supplemental Digital Content 7, http://links.lww.com/MS9/A117). The tricuspid valve is of normal morphology, without exploitable regurgitation with a slight pulmonary leak allowing an average pulmonary arterial pressure to be estimated at 37+10=47 mmHg. The left ventricle is dilated (LV end-diastolic diameter at 65 mm, LV systolic diameter at 40 mm) hypertrophied (end-diastolic interventricular septum and end-diastolic posterior wall at 20 mm) and a LV *ejection fraction* at 64%. The inferior vena cava and the supra-hepatic veins were dilated. There was also a circumferential pericardial effusion measuring 28 mm posterior to the LV, 13 mm anterior to the right ventricle without any signs of compression. The laboratory work-up found a biological inflammatory syndrome with a C-reactive protein at 111 mg/l, hyperleukocytosis of 17 210 elements/mm^3^ w, microcytic hypochromic anemia with a hemoglobin level of 10 g/dl, normal ferritinemia, a platlets 329 000 elements/mm^3^. Other results are summarized in (Table 1).The diagnosis of IE was suspected, and three blood cultures were taken at 1 h intervals, all positive for enterococci. As part of the extension workup, a thoracoabdominal and brain computed tomography scan with contrast injection was performed showing a hypodense right frontal cerebral spot with poor limits, not enhancing after injection of contrast medium in favor of an ischemic stroke (Fig. 3A) an aortic coarctation downstream of the left subclavian artery (Fig. 3F) images of mycotic aneurysms of the descending aorta (Fig. 3B), right splenic and renal infarcts (Fig. 3C, D, E). The fundoscopic examination showed Roth spots in both eyes (Fig. 1B). The immunological workup; rheumatoid factor, C3 and C4 complement, and 24 h proteinuria were all negative.

**Table 1:** Significant laboratory findings

Albumin	35.00	34–54
C-reactive protein (mg/l)	111	6–12
Urea (g/l)	0.22	<0.45
Creatinine (mg/l)	9	(6–12)
Potassium (mmol/l)	4.2	(3–5)
Natremia (mmol/l)	139	(135–140)
White blood cells (E/mm^3^)	17 210	(4000–10 000)
neutrophilic polynuclear	15 820	(2000–7500)
Hemoglobin (g/dl)	10	>13
Hematocrit	51.2	40–52
Platlets	329 000	(150 000–400 000)
Feriitin (ug/l)	95	(30–300)
*Procalcitonin*(ug/l)	0.7	<0.45

The diagnosis of a confirmed IE is retained, and the patient was treated by antibiotics therapy based on amoxicillin 12 g/day (200 mg/kg/d) gentamycin 160 mg/day, then referred to the department of cardiovascular surgery where he received a surgical cure of the coarctation of the aorta, an aortic valve tube, and a replacement of the mitral valve, which was done successfully (video 7, Supplemental Digital Content 8, http://links.lww.com/MS9/A118 and 8, Supplemental Digital Content 9, http://links.lww.com/MS9/A119). The postoperative evolution was favorable.

## Discussion

IE is an infection of the endocardium by a microorganism circulating in the bloodstream, most often bacterial, with the formation of vegetation. More rarely, this invasion may be localized at the level of valve prostheses or intracardiac materials. The incidence of IE is estimated to be 30 cases per million people per year^2^. Recent epidemiological studies report an increase in the incidence of AR with age, with a peak between 70 and 80 years of age, and a male predominance with a male/female ratio greater than 3/1^3^. Despite medical and surgical progress, it remains a serious pathology, with an estimated 20% in-hospital mortality and 40% at 5 years^4^.

IE on congenital heart disease was found in 6.8% of cases, of which BAV is the most frequent congenital heart disease, according to Osler’s old series, According to the series of Michelena and Tzemos, the incidence of endocarditis in BAV is 1.8–2% of patients^5^, but these endocarditis have the characteristic to be severe.

The enterococcus isolated from our patient was not the most frequently isolated bacterium in simple or complicated endocarditis; it ranked third after staphylococcus and streptococcus^6^.

IE can be the cause of *several* extracardiac manifestations, through vascular and/or immunological phenomena. Rothe’s spot has been found in 5% of cases, mucocutaneous manifestations (purpura, false Osler’s panic, Janeway’s erythema, conjunctival hemorrhages) also in 5% of cases, and seem to be associated with a higher risk of extracardiac complications^7^.

Embolic complications found in our patients were the most frequent extracardiac complications of IE (30– 50%), they are secondary to septic emboli from vegetations and are more frequent with left heart IE^6^. Septic emboli can affect all viscera, and be the origin of infarction or secondary infectious localization. The usual embolic localizations are cerebral (ischemic stroke, meningitis), splenic, or renal (abscess or infarction). The risk factors for the occurrence of embolic migration are the large size of the vegetation (>10–15 mm), vegetation in mitral position and mobile, embolic history, IE with staphylococcus aureus, or enterococcus^8,9^.

Neurological complications occur in 30% of cases and represent the second cause of mortality after cardiac complications. Various and sometimes interrelated mechanisms: Ischemic following a septic embolism, it involves the territory of the middle cerebral artery in over 90% of cases. It is observed two to three times more often in mitral IEs than in aortic IEs. Hemorrhagic by rupture of a mycotic aneurysm Infectious: purulent meningitis, abscess, or meningoencephalitis^10,11^.

Mycotic aneurysms are a classic complication of streptococcal, and may remain asymptomatic or cause serious complications^12^. Their mechanism is multifactorial: deposition of immune complexes in the arterial wall or direct septic damage to the wall.

Cardiac surgery is performed in 50% of patients who develop IE. There are three indications for surgery: acute heart failure related to severe valvular insufficiency, valvular obstruction, fistula, or shock (cardiogenic / septic). An infection not controlled by antibiotic treatment. Embolic indications in the case of vegetation with high emboligenic potential^2^.

## Conclusion

The prognosis of IE is worsened by the addition of cardiac and extracardiac complications such as mycotic aneurysms and septic embolic events.

Cardiac surgery is performed in 50% of patients in association with a well-conducted antibiotic therapy in case of IE with complications to which medical treatment is insufficient.

## Ethical approval

NA.

## Consent

Written informed consent was obtained from the patient for publication of this case report and accompanying images. A copy of the written consent is available for review by the Editor-in-Chief of this journal on request.

## Sources of funding

No funding was received for this work.

## Author contributions

N.E.O.: project administration; N.I.: conceptualization and supervision; O.D., O.E.L. A.: data collection: data analysis; M.B.: writing – original draft; C.D.A.: review and editing.

## Conflicts of interest disclosure

The authors have no competing interests to declare that are relevant to the content of this article.

## Research registration unique identifying number (UIN)

NA.

## Guarantor

Mohammed Boutaybi.

## Provenance and peer review

Not commissioned, externally peer-reviewed.

## Supplementary Material
