# Supplementary figures and images for: Mitro-aortic infective endocarditis on bicuspid aortic valve multicomplicated: a case report
Source: Ann Med Surg (Lond). 2023 May 3;85(6):3017–21. doi: 10.1097/MS9.0000000000000745 (PMC10289630; doi:10.1097/MS9.0000000000000745)

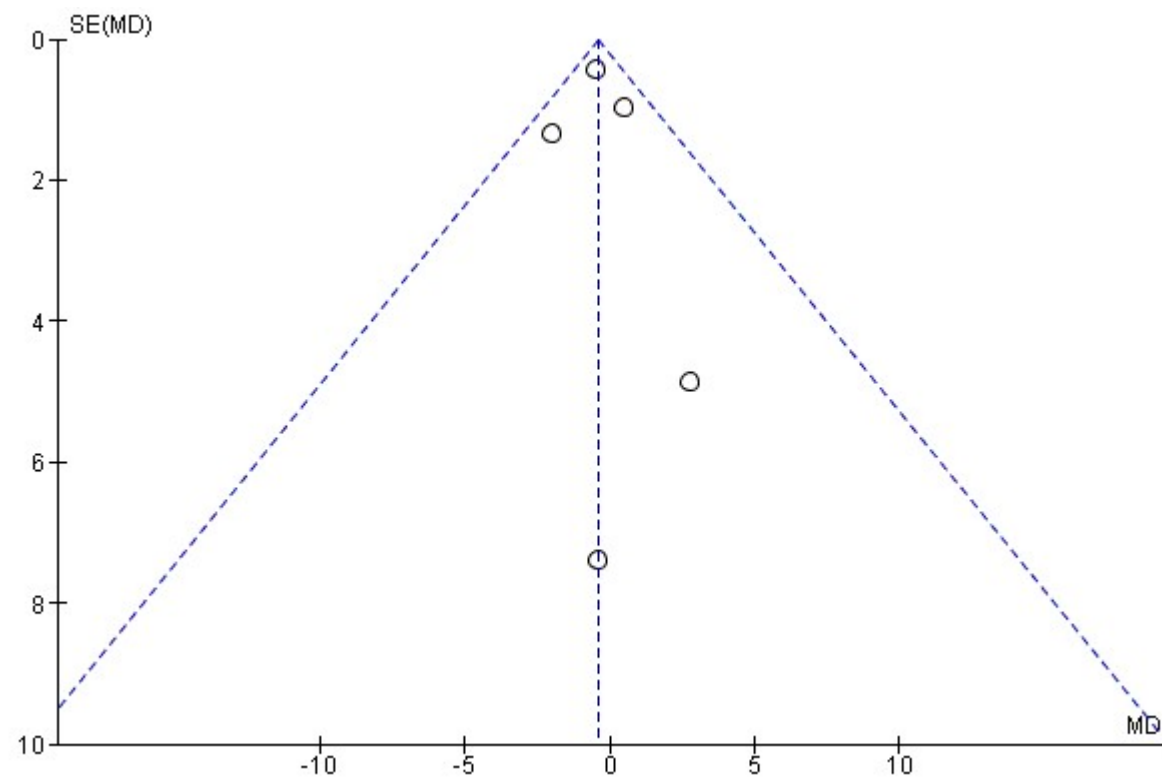

Supplement: Supplementary file 3 [file ms9-85-3017-s003.pdf]

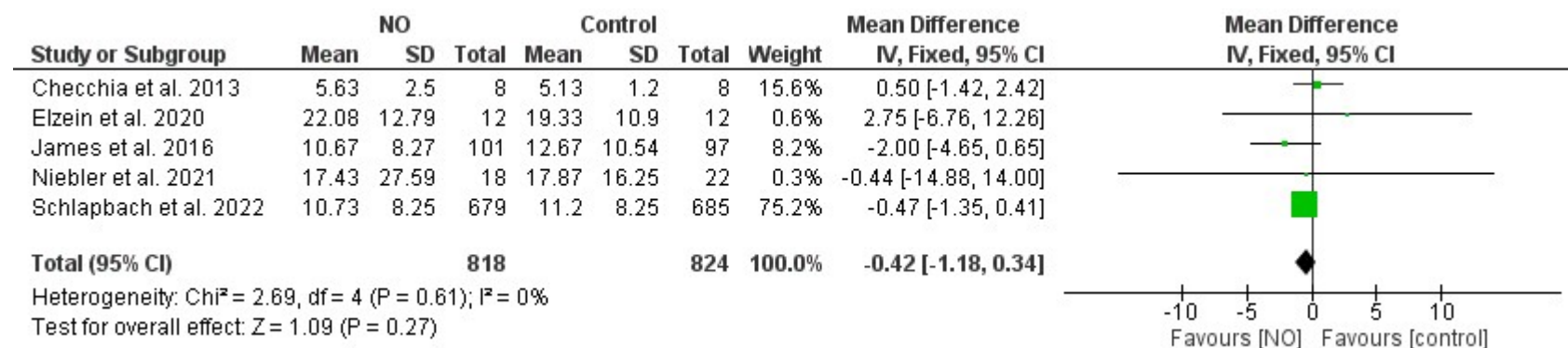

Supplement: Supplementary file 4 [file ms9-85-3017-s004.pdf]

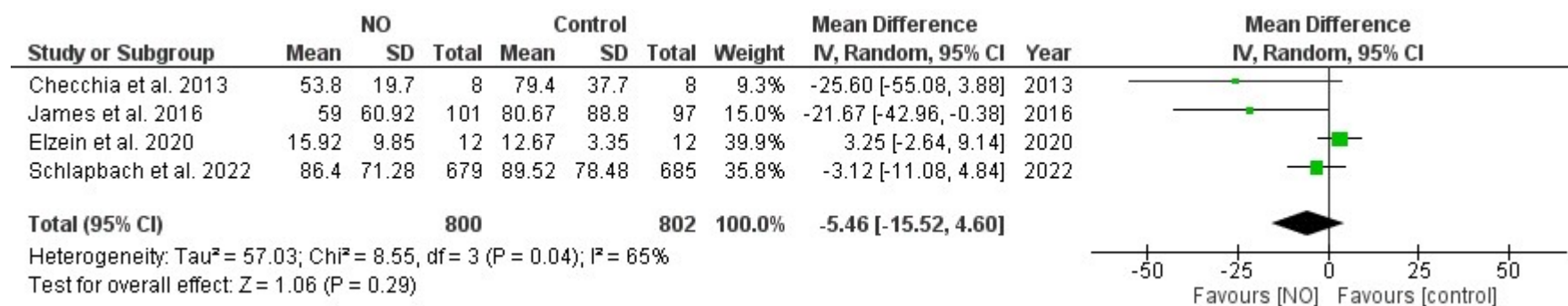

Supplement: Supplementary file 5 [file ms9-85-3017-s005.pdf]

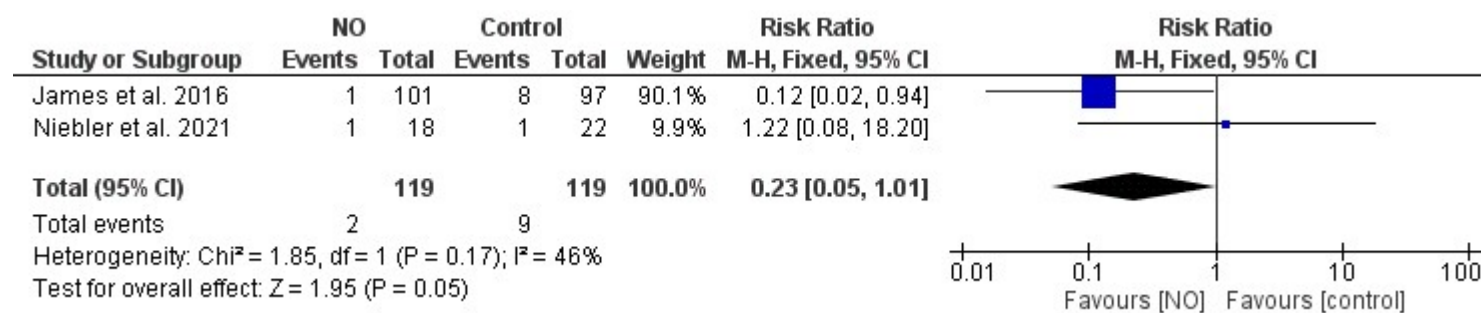

Supplement: Supplementary file 6 [file ms9-85-3017-s006.pdf]

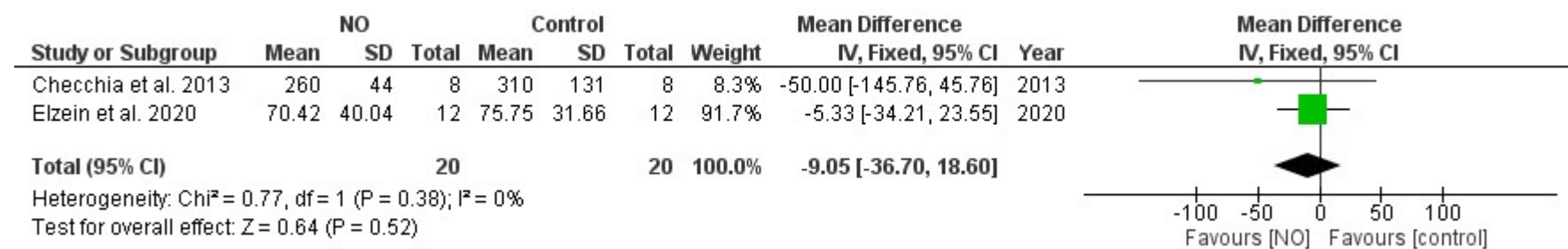

Supplement: Supplementary file 7 [file ms9-85-3017-s007.pdf]

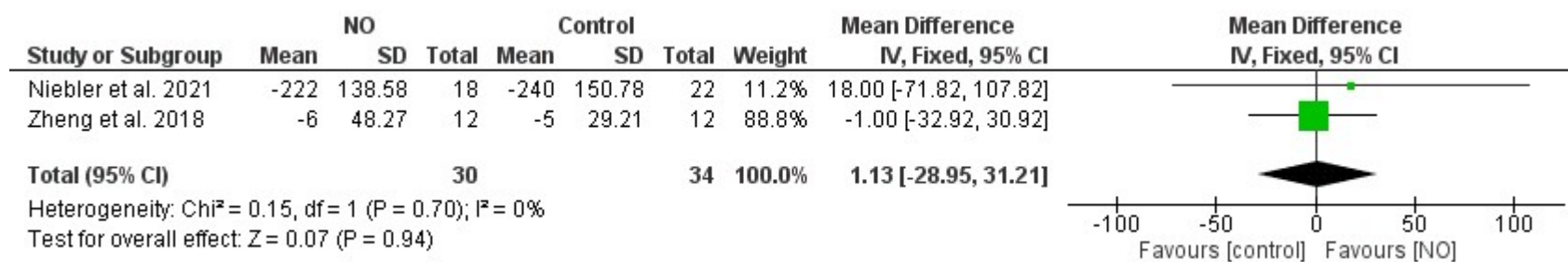

Supplement: Supplementary file 8 [file ms9-85-3017-s008.pdf]

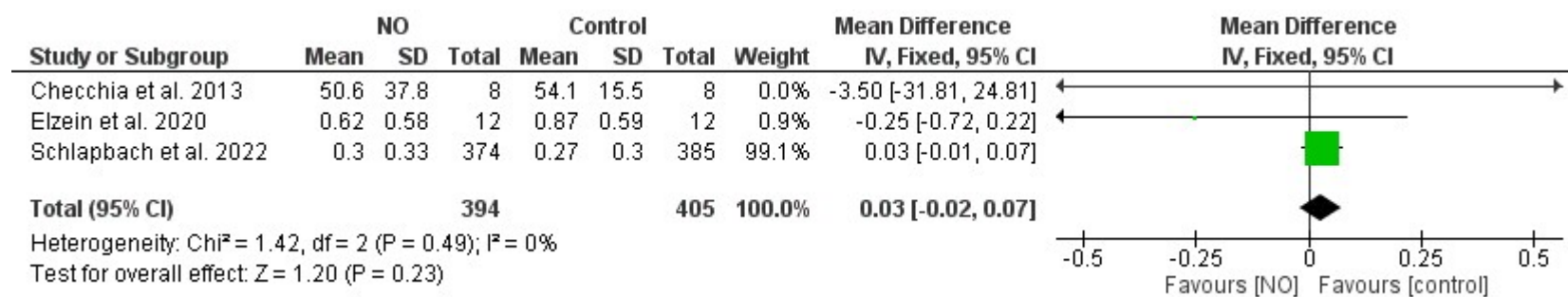

Supplement: Supplementary file 9 [file ms9-85-3017-s009.pdf]

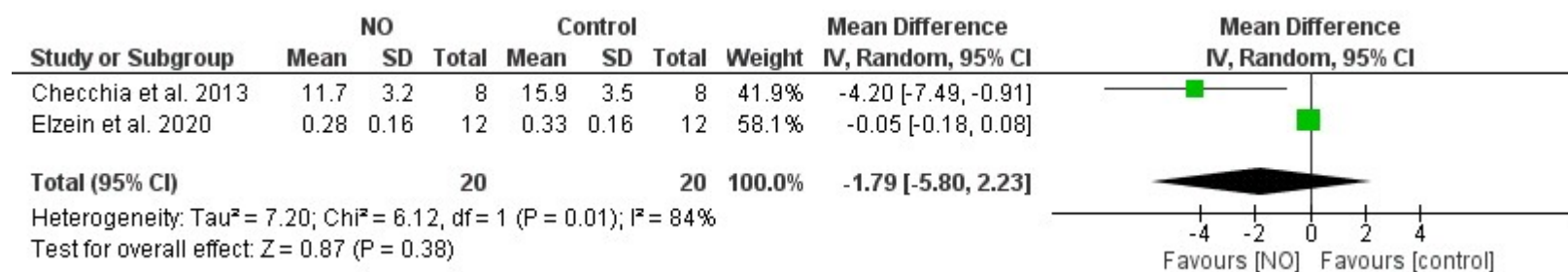

Supplement: Supplementary file 10 [file ms9-85-3017-s010.pdf]

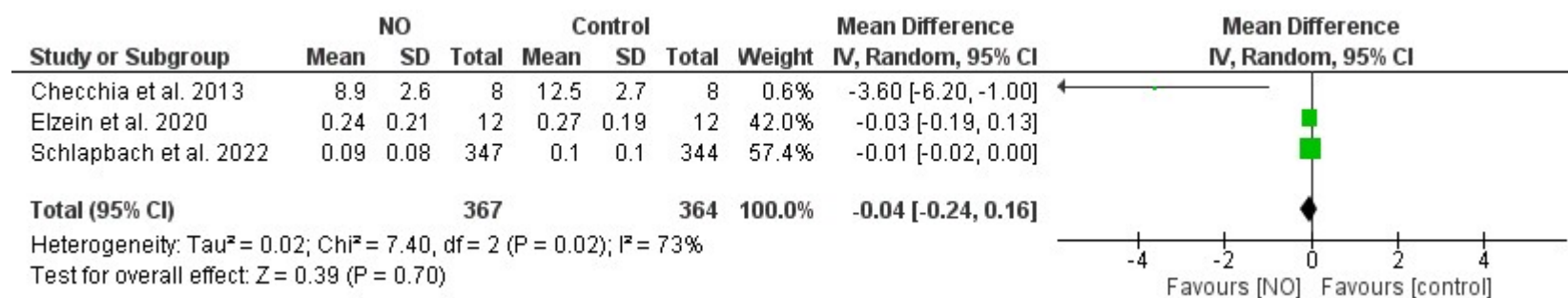

Supplement: Supplementary file 11 [file ms9-85-3017-s011.pdf]

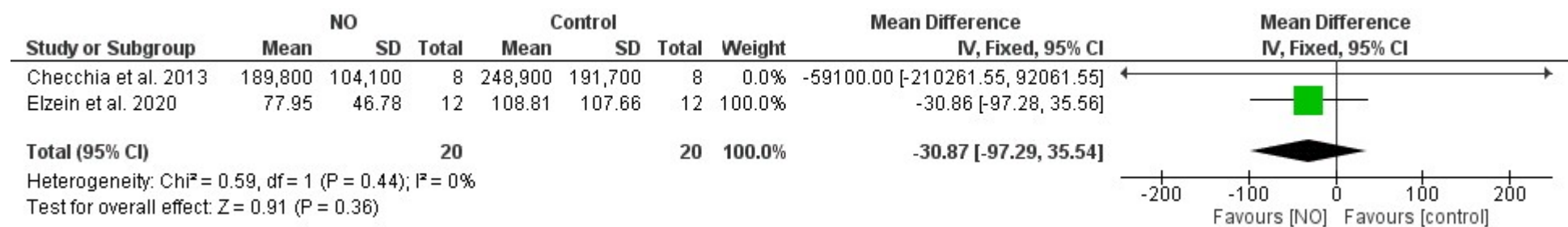

Supplement: Supplementary file 12 [file ms9-85-3017-s012.pdf]

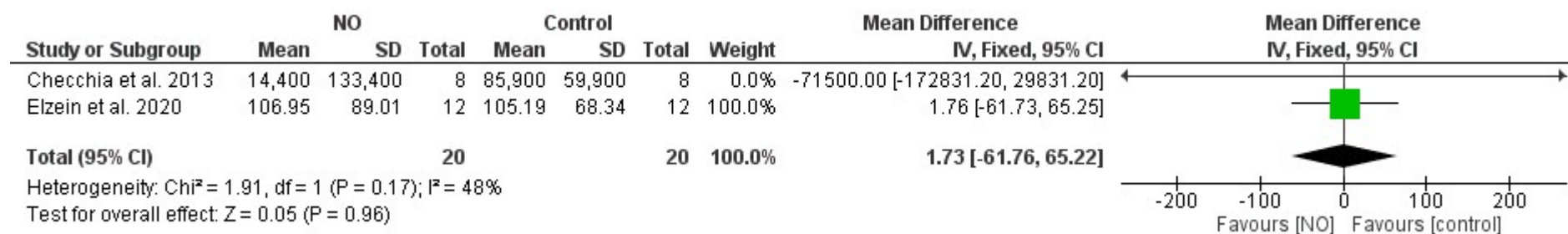

Supplement: Supplementary file 13 [file ms9-85-3017-s013.pdf]

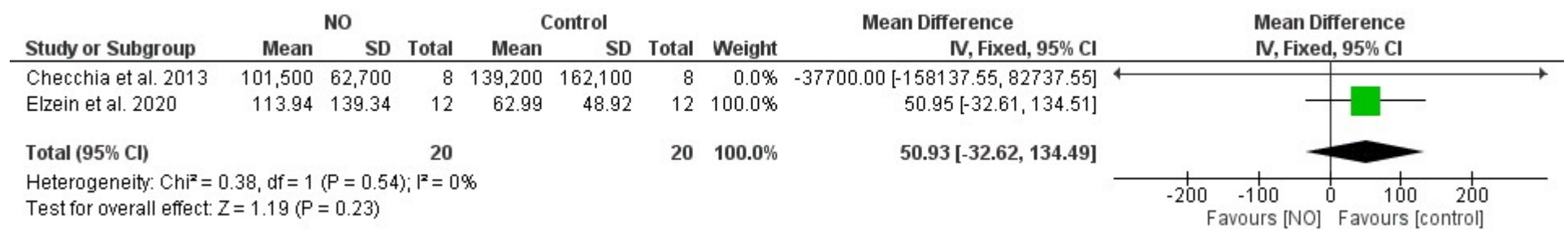

Supplement: Supplementary file 14 [file ms9-85-3017-s014.pdf]
