# Supplementary material for: Mitro-aortic infective endocarditis on bicuspid aortic valve multicomplicated: a case report
Source: Ann Med Surg (Lond). 2023 May 3;85(6):3017–21. doi: 10.1097/MS9.0000000000000745 (PMC10289630; doi:10.1097/MS9.0000000000000745)
Supplement: Supplementary file 15 [file ms9-85-3017-s015.docx]

**1 Figure legends**

**Figure S.1:** Funnel plot of the included studies.

**Figure S.2:** Forest plot of the analysis length of hospital stay (days).

**Figure S.3:** Forest plot of the analysis length of ICU Stay (hours).

**Figure S.4:** Forest plot of the analysis need for ECMO within 48 hours.

**Figure S.5:** Forest plot of the analysis chest tube output within 48 hours (mL).

**Figure S.6:** Forest plot of the analysis postoperative change in platelets count (*10^9^/L).

**Figure S.7:** Forest plot of the analysis cardiac troponin level (ng/ml).

**Figure S.8:** Forest plot of the analysis IL-8 level (pg/ml).

**2 Figures**

**Figure S.1:**

**
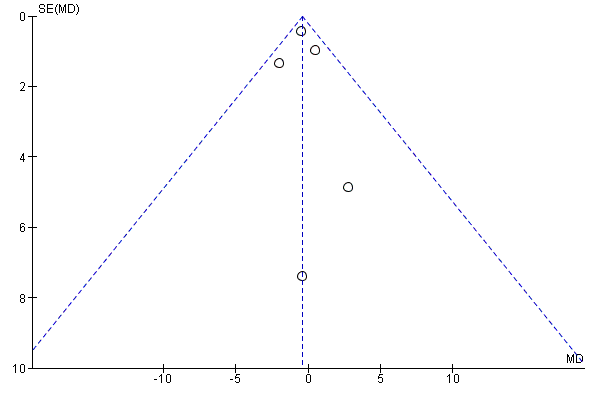
**

**Figure S.2:**

**
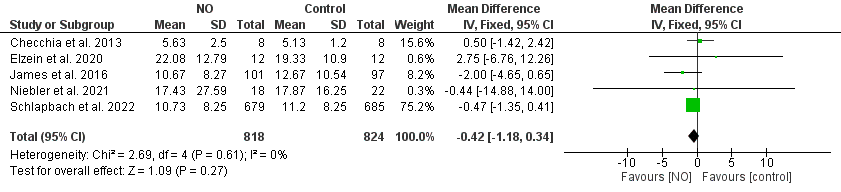
**

**Figure S.3:**


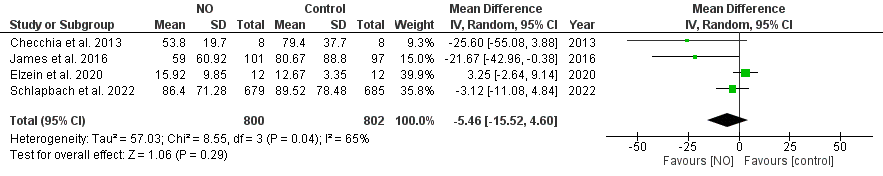


**Figure S.4:**


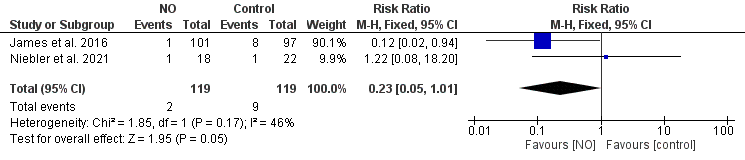


**Figure S.5:**


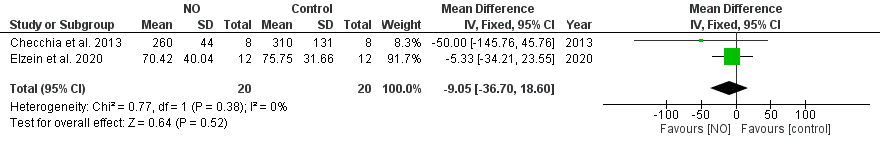


**Figure S.6:**


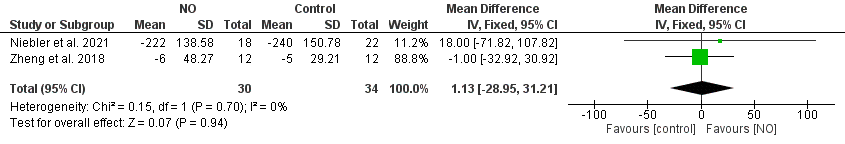


**Figure S.7:**

**
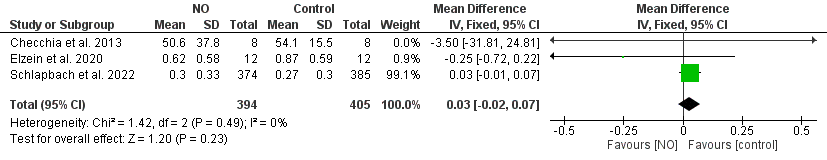
**

***a***

**
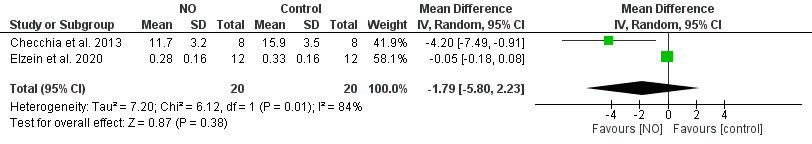
**

***b***

**
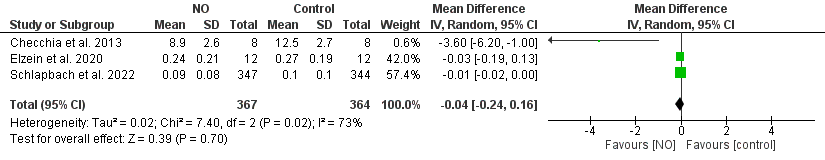
**

***c***

**Figure S.8:**


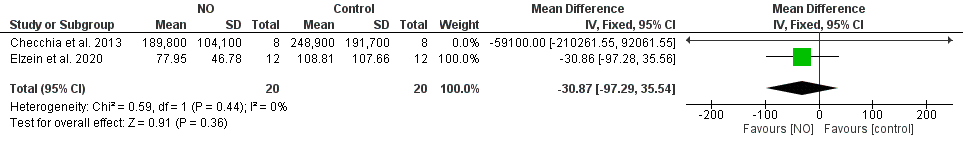


***a***

**
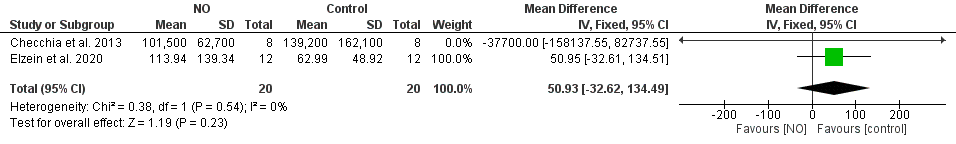
**

***b***

**
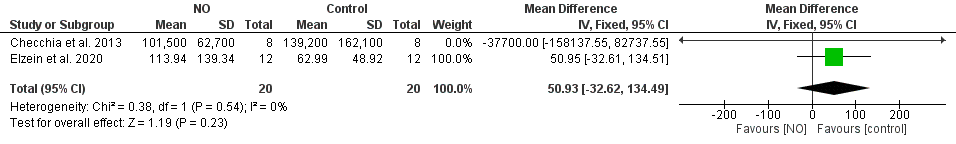
**

***c***
